# Lateral Extra-Articular Tenodesis Does Not Impact Postural Stability After Pediatric Anterior Cruciate Ligament Reconstruction with Hamstrings Tendons

**DOI:** 10.3390/medicina62050857

**Published:** 2026-04-30

**Authors:** Alex Delisle, David Mazy, Siyu Wang, Zoé David, Mélanie Sarda, Guy Grimard, Marie-Lyne Nault

**Affiliations:** 1 Faculty of Medicine, University of Montreal, 2900 Bd Edouard-Montpetit, Montréal, QC H3T 1J4, Canada; 2 Surgery Department, Sainte-Justine University Hospital Centre, 3175 Chemin de la Côte-Sainte-Catherine, Montréal, QC H3T 1C5, Canada; 3 Physical Therapy Department, Sainte-Justine University Hospital Centre, 3175 Chemin de la Côte-Sainte-Catherine, Montréal, QC H3T 1C5, Canada

**Keywords:** anterior cruciate ligament reconstruction, lateral extra-articular tenodesis, Biodex stability system, postural balance, rehabilitation, pediatric, hamstring autograft

## Abstract

*Background and Objectives*: Given the high rates of anterior cruciate ligament graft rupture in the pediatric population, lateral extra-articular tenodesis (LET) is increasingly used in combination with anterior cruciate ligament reconstruction (ACLR) to mitigate the risk of re-injury. This study aimed to compare postoperative postural stability between patients undergoing ACLR with and without LET. It was hypothesized that postural stability would be comparable between patients with and without LET. *Materials and Methods*: This retrospective, single-center, double-surgeon case–control study included patients who underwent primary ACLR using hamstring tendon autografts between January 2022 and May 2025. Postoperative postural stability was assessed using the Biodex Stability System (BSS) global stability index (GSI), which was the primary outcome of interest. Demographic and surgical data were collected as well as all postoperative GSIs. GSI comparisons between the LET and no-LET groups were made at ≤6 months and >6 months postoperatively. Secondary analysis compared GSI differences between the healthy and operated legs. *Results*: Among 229 patients screened, 100 met the inclusion criteria (median age, 16 years [IQR, 15–17]); 65 underwent LET and 35 did not, and 54 were female (54%). The groups were comparable on demographic and surgical data (*p*-value: n.s.). No operated leg GSI difference was observed between the LET and no-LET groups at ≤6 months (*p* = 0.372) and >6 months postoperatively (*p* = 0.424). Patients with LET had significantly better (lower) healthy leg GSIs (Mean ± SD; 2.7 ± 0.9) than no-LET patients (3.9 ± 1.8) at >6 months postoperatively (*p* = 0.004). At ≤6 months, patients showed better GSIs on their operated limb (Median [IQR]; 2.6 [2.1–3.9]) compared to the healthy limb (3.5 [2.3–4.6]) (*p* = 0.003). This difference disappeared at the latest follow-up. *Conclusions*: The addition of LET concomitant with ACLR was not associated with a significant difference in postural stability, as assessed by the GSI from the BSS. However, given the sample size and study limitations, these findings should be interpreted with caution. Increased attention to the healthy limb during ACLR rehabilitation may be warranted, particularly in the early postoperative period (<6 months). Further studies with larger cohorts are needed to confirm these observations.

## 1. Introduction

Anterior cruciate ligament (ACL) rupture is an increasingly common injury among young athletes [1]. Graft failure rates are also more prevalent in this population, highlighting the need for new strategies to reduce this complication [2,3]. The growing popularity of lateral extra-articular procedures (LEAP) performed concurrently with ACL reconstruction (ACLR), particularly in higher-risk populations such as young patients, those returning to pivoting sports, and revision cases, has been associated with a substantial reduction in re-rupture rates [4,5]. LEAPs include a range of procedures, such as anterolateral ligament (ALL) reconstruction and lateral extra-articular tenodesis (LET), including modified Lemaire or McIntosh techniques [6]. LEAPs are performed on both adults and children to reduce rotational instability [7,8].

Although still debated, several indications are recognized for LEAP, including young age, significant rotational instability assessed by a grade two or higher pivot shift, hyperlaxity, and return to contact or pivoting sports [9,10,11]. More recently, expert recommendations have suggested performing LET in all active pediatric patients [10,11].

This procedure offers additional advantages compared to ACLR alone. The systematic review by Zabrzyński et al. demonstrated improved clinical outcomes, including lower graft rupture rates, reduced residual pivot and laxity, and better overall knee stability, without an increased risk of long-term osteoarthritis [7]. Numerous studies have reported improved postoperative stability when LEAP is performed in both adult and pediatric populations [12,13]. Importantly, these benefits appear to occur without compromising muscle recovery, functional test performance, or return to sports outcomes [12,14,15,16,17].

Postural stability is essential for sports performance following ACLR, although it is frequently impaired after surgery [18,19,20]. This instability has also been associated with an increased risk of second ACL injury, highlighting its importance in rehabilitation and return-to-sport decision-making [21]. The Biodex Stability System (BSS) allows for the quantitative assessment of postural stability through the global stability index (GSI), calculated by the device’s internal software [22]. This tool has been widely used in various clinical populations, including patients with chronic ankle instability, lower limb amputation, non-specific low back pain, cerebral palsy, and visual impairment [23,24,25,26,27].

The BSS is also used after ACLR to assess postural stability [28]. Under static conditions, this tool measures the deviation of the center of pressure, while in dynamic conditions, it measures the degree of tilt [23]. To the best of our knowledge, no studies have examined the impact of performing a LET in addition to ACLR on GSI in either adults or children. The objective of this study is to evaluate the impact of the LET on GSI post-ACLR with hamstring tendon graft in a pediatric population. A secondary objective is to compare the healthy leg and operated leg GSIs. The hypothesis is that adding a LET would not negatively affect GSIs and that the healthy leg will have better indexes than the operated leg.

## 2. Materials and Methods

### 2.1. Ethical Approval

This retrospective study was conducted at the Sainte-Justine University Hospital Center. It was performed in accordance with the Declaration of Helsinki and approved by the institution’s Ethics Committee (protocol code 2026-9093; approval date: 22 January 2026)

### 2.2. Study Design and Population

A retrospective analysis was performed on a consecutive series of ACLRs performed by two orthopedic surgeons specializing in pediatric sports medicine at Sainte-Justine University Hospital (pediatric tertiary center).

The inclusion criteria comprised patients aged 20 years or younger who underwent primary ACLR using hamstring tendon autografts and for whom bilateral BSS scores were available. Patients with concomitant meniscal injuries were included regardless of whether surgical treatment was performed. Patients who underwent multiligament knee reconstruction or ACLR using grafts other than hamstring tendons were excluded. Hamstring tendon autografts represent the standard graft choice at the treating institution, and alternative graft types are rarely used. These patients were therefore excluded to ensure cohort homogeneity and to minimize potential selection bias related to graft choice. Cartilage lesions requiring repair procedures were excluded; however, no such cases were identified in the present cohort.

### 2.3. Surgical Technique

All surgical procedures were performed by two fellowship-trained sports orthopedic surgeons. ACLR was performed using a hamstring tendon autograft. The semitendinosus was harvested first; if the quadrupled graft diameter was <8 mm, the gracilis tendon was also harvested and combined in a total 6-strand construct to achieve a final graft diameter of 8–10 mm. The femoral tunnel was performed with an inside-out technique through the anteromedial portal, and the tibial tunnel was positioned to be in the center of the tibial remnant. All ACLRs were carried out with an inside-out technique using femoral and tibial adjustable-loop cortical buttons (Infinity, ConMed, Utica, NY, USA). The LET was performed according to the modified Lemaire technique [29]. A 1 cm × 8 cm strip of the posterior iliotibial band was harvested while preserving its distal attachment to the Gerdy tubercle. After ACL fixation, the strip was passed below the lateral collateral ligament and fixed to the femur with a suture anchor (TruShot, ConMed) at 45° of knee flexion in neutral rotation.

### 2.4. Rehabilitation Protocol

Following surgery, the focus was on restoring knee range of motion, reducing swelling, and reactivating the muscles while protecting the graft. According to the institutional protocol, the objective was to achieve full knee extension and 90° of flexion within the first two weeks and >120° of flexion by approximately six weeks, with progression guided by symptoms and functional capacity. Isokinetic strength testing was first performed 4 months post-ACLR and repeated every 4 to 6 weeks until limb symmetry was achieved (<10% interlimb difference). During early rehabilitation stages, physiotherapists of the treating institution primarily focus on training the operated leg. Cross-education effects were not specifically targeted but may have occurred as a result of unilateral strength training. Once this isokinetic symmetry was reached, patients then proceeded to bilateral and functional exercises in preparation for the return to sport. These exercises include bipodal strengthening exercises such as squats and leg press, as well as neuromuscular and balance training (e.g., deep squat progression, Y-Balance test, and dynamic stability exercises). Using their clinical judgment, physiotherapists then used hop tests and BSS assessments. While patients needed to have passed their isokinetic tests to start hop tests, the BSS assessment could be performed throughout rehabilitation, even before leg symmetry was achieved. The GSI that the BSS assessment provides was used as complementary information but not as a standalone criterion. Complete return to sport was usually made when patients passed their hop tests (<10% interlimb difference). All patients included in the present study underwent the same standardized rehabilitation protocol.

### 2.5. Biodex Stability System

**Figure 1:**
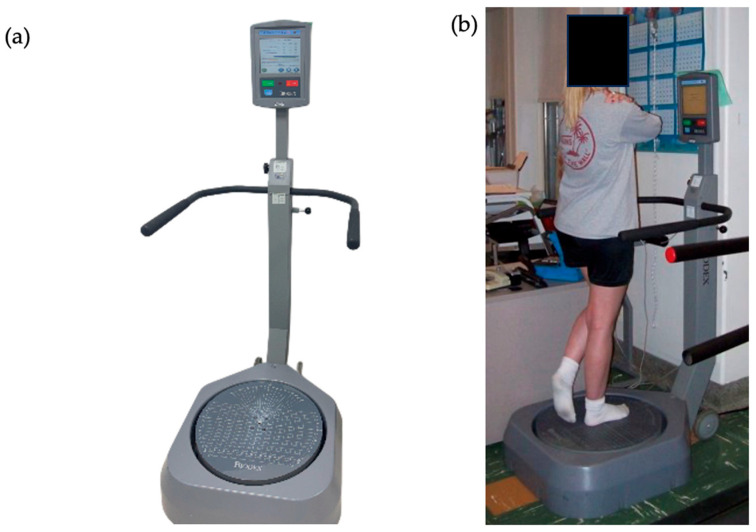
(**a**) Biodex Stability System setup. (**b**) With patient testing right leg.

The BSS (Biodex Medical Systems, Shirley, NY, USA) is a movable multiaxial balance platform on which patients stand on one leg. It provides up to 20° of surface tilt in all directions and allows adjustment of stability levels ranging from 1 (least stable) to 12 (most stable) [24]. The platform is interfaced with proprietary Biodex software(version 3.x) for data acquisition and analysis (Figure 1).

For sports rehabilitation, physiotherapists of the treating institution set the platform stability to level 4. The test was routinely performed during follow-up outpatient assessments. Patients removed their shoes before stepping on the platform. They started the test on their healthy leg for security purposes. Once the centering process was completed, patients were instructed not to move their weight-bearing leg. They were encouraged to bear weight on the other foot until the start of the test. Before official testing, the physiotherapist would release the platform and let the patient familiarize himself with the task for 15 s, to then stabilize the platform again. Once the test started, the platform released, and the physiotherapist hid the screen showing the center of pressure. Patients then had to maintain balance for 20 s on the unstable platform, with their arms crossed on their shoulders and their non-weight-bearing leg in the air, away from the other one, with their eyes open. The platform stabilized automatically when the test ended. A 10 s break where patients could weight-bear followed. Two more 20 s blocks were completed in the same manner. The GSI was calculated directly by the BSS computer after patients completed the three 20 s tests. The same protocol was completed immediately after for the operated leg. Lower GSI represents more stable patients, while higher GSI indicates instability [22]. Theoretically, the lowest possible GSI is zero. The GSI corresponds to a composite score between the anteroposterior and mediolateral scores calculated by the system. It corresponds to the amount of tilt that occurred in both the frontal and sagittal planes [22].

In the treating institution, pediatric athletes usually perform the test throughout their rehabilitation until they obtain a GSI of around 2.0–2.5 on their operated leg. No pediatric-specific normative thresholds have been formally established. Only adequately trained physiotherapists conducted BSS assessments on patients. They all followed the same exact procedure described. Available postoperative GSIs were separated in two periods for analysis: before and after 6 months. A subset of patients had measurements in both time periods; therefore, these groups were not mutually exclusive, and their sample sizes were not additive.

Although a standardized protocol was followed by the two trained physiotherapists, certain factors such as inadvertent external support (e.g., hand contact with the railing or contralateral limb contact) were not formally controlled or recorded.

### 2.6. Data Collection and Analyses

ACL rupture was diagnosed based on clinical examination and confirmed by magnetic resonance imaging. Demographic data such as age, gender, and body mass index were extracted from electronic medical records. The same applied to surgical data, such as laterality, concomitant LET, meniscus tears, meniscus surgery, ACL graft diameter, and pre-operative pivot shift. Preoperative status was assessed clinically, including pivot shift grading. Three different subjective grades apply to a positive pivot shift test to assess dynamic laxity: grade I (glide), grade II (clunk), and grade III (explosive) [30].

All postoperative BSS GSI were extracted from patients’ electronic medical records. The primary outcome measure was the GSI from the BSS.

Comparisons were performed between patients who underwent ACLR without LET and those who underwent ACLR combined with LET. Analyses were completed for early (≤6 months) and late (>6 months) rehabilitation periods. The six-month threshold was retained due to the progressive introduction of pivoting without contact at that time point. Secondary analyses compared balance outcomes between the operated limb and the contralateral healthy limb.

### 2.7. Statistical Analysis

All statistical analyses were performed using IBM SPSS Statistics (version 30, IBM Corp., Armonk, NY, USA). Descriptive statistics were calculated for continuous variables, and frequencies for categorical variables. The normality of continuous variables was assessed using the Shapiro–Wilk test for groups with and without LET.

Independent *t*-tests and the non-parametric Mann–Whitney U test were used to assess whether there were significant differences between the groups with and without LET, for normally distributed and non-normally distributed variables, respectively.

For categorical variables, the chi-square test was used to assess whether there was a difference between the groups with and without LET. Fisher’s exact test was used when the number of variables was fewer than five. The Mann–Whitney U test was used for ordinal variables.

GSIs calculated by the BSS were compared between the LET and non-LET groups using parametric or non-parametric tests depending on normality (independent samples *t*-tests and Mann–Whitney U test). When multiple BSS assessments were available within the same time window, the test demonstrating the best performance on the operated limb was retained (lowest GSI) in order to reflect the participant’s maximal functional capacity during that period. Comparisons between the healthy and operated legs were performed using paired *t*-tests or the Wilcoxon Signed Rank Test for related samples, depending on whether the differences were normally distributed or not, respectively. A *p*-value <0.05 was considered statistically significant.

Because no minimal clinically important difference (MCID) has been established for the GSI, an exploratory distribution-based estimate was calculated using the 0.5 standard deviation method.

## 3. Results

**Figure 2:**
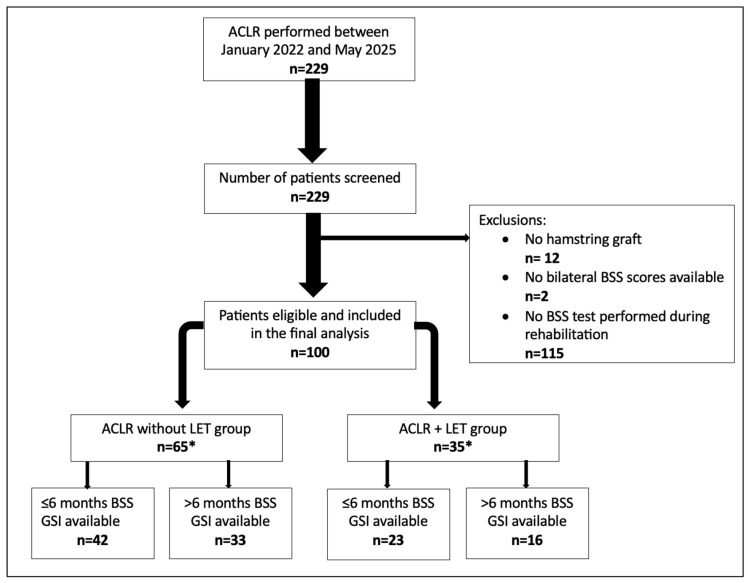
Study participants flowchart. ACLR, anterior cruciate ligament reconstruction; BSS, Biodex Stability System; LET, lateral extra-articular tenodesis; GSI, Global Stability Index. * The total number of patients across subgroups exceeds the overall sample because some patients contributed data at both time points.

A total of 229 consecutive patients were screened for eligibility, of whom 100 met the inclusion criteria (Figure 2). Patient demographic and surgical information are presented in Table 1. No demographic or surgical variables differed significantly between the LET and no-LET groups (all *p*-values non-significant), supporting the comparability of the study groups (see Table 2).

Table 3 shows the mean or median GSI depending on the leg assessed for the LET and non-LET groups for the two time periods.

Regarding the healthy leg, the LET group showed a significantly lower GSI score more than 6 months after surgery compared to the non-LET group (mean difference 1.15; 95% CI, 0.39–1.91; *p* = 0.004). No other significant differences (*p* > 0.05) were found between groups for the other time points or the operated leg (Table 3). Based on the pooled standard deviation of the healthy leg scores after 6 months (SD ≈ 1.52), the exploratory MCID estimate was approximately 0.76 points, suggesting that the observed difference exceeds this threshold. Table 4 presents the comparison of the GSI between the healthy and operated legs across postoperative time periods, using the Wilcoxon signed-rank test. Table 5 shows that the excluded group differs from the group included on two variables: median age and median BMI.

Linear regression analysis showed no significant association between surgery date and GSI for the healthy leg (β = −0.164, *p* = 0.102) and the operated leg (β = −0.006, *p* = 0.950). A post hoc power analysis was conducted at a significance level of α = 0.05 to evaluate whether the available sample size was sufficient to detect a difference in GSI between patients with and without LET. Based on the observed proportions, the analysis demonstrated a statistical power of 71%, indicating a moderate probability of detecting the observed association.

## 4. Discussion

The most important finding of this study is that the addition of a LET procedure to ACLR with hamstring tendon autograft in pediatric patients does not significantly alter GSI before and after 6 months post-surgery. Clinically, this suggests the additional tenodesis does not affect dynamic stability during rehabilitation.

An important rationale for performing a LET is to control rotational instability [7]. During BSS testing, the evaluated limb remains stationary on an unstable platform, which minimizes rotational or pivoting movements. This testing condition may therefore explain why no significant differences were observed between groups. Additionally, the BSS test may primarily reflect the dynamic neuromuscular control and postural stability provided by periarticular muscle activation rather than isolated ligamentous constraints or additional anterolateral procedures such as LET.

Importantly, the absence of impaired postural stability following LET is reassuring, particularly given that it is an additional surgical procedure. These findings support that LET can be safely performed in the pediatric population without negatively affecting postural control during rehabilitation. This is consistent with existing literature reporting that LET is a safe adjunct procedure in pediatric ACLR with no additional long-term complications [7,10,11].

Across all conditions (before and after the six-month postoperative mark, healthy and operated legs), the LET group consistently displayed lower mean and median GSI scores, indicating superior stability. However, only the healthy leg GSI showed a statistically significant difference beyond 6 months postoperatively. Before 6 months postoperatively, results showed a trend favoring the LET group regarding healthy leg GSIs, although this difference was not statistically significant. Beyond the 6-month mark, the healthy leg GSI was significantly lower (better) in the LET group compared to the no-LET group. No MCID has previously been established for the GSI. Using a distribution-based approach (0.5 SD), the estimated MCID in this cohort was approximately 0.76 points. The observed difference of 1.15 points exceeds this threshold, suggesting that the difference may be clinically meaningful, although this estimate should be interpreted cautiously and confirmed in future studies.

A possible hypothesis for this unique difference can be the cross-education effect induced by intensive rehabilitation of the operated limb, which may enhance postural control in the contralateral healthy limb. The cross-education effect refers to strength and neuromuscular adaptations in the contralateral limb following unilateral training, primarily mediated by neural mechanisms [31]. It has been described during early phases of ACLR rehabilitation, where concentric and eccentric quadriceps strengthening of the healthy limb improved recovery of the operated limb compared to controls [32]. The literature therefore supports that the difference in healthy leg GSI observed beyond 6 months in the LET group may be related to this phenomenon, although it remains a hypothesis in the current study.

Interestingly, patients had better GSI values on their operated leg compared to their healthy leg before 6 months postoperatively. However, the difference disappeared after the 6-month mark. This result is interesting, as interlimb asymmetries in functional return-to-sport tests after ACLR usually favor the healthy limb [33,34]. One possible explanation is the rehabilitation protocol used in the treating institution, where early rehabilitation focuses primarily on the operated limb until symmetry is achieved on isokinetic testing (<10% interlimb difference). This focus may account for the observed differences before 6 months postoperatively. As rehabilitation progresses, training of the healthy limb is progressively introduced, which may reduce interlimb differences over time. In addition to high graft rupture rates, studies have demonstrated important contralateral injury rates in the pediatric population [35]. It is therefore important not to neglect the healthy limb during rehabilitation, particularly in this population. Education of young patients regarding this issue is required. Nevertheless, the initial focus on the operated limb does not appear to impact outcomes beyond 6 months postoperatively, as interlimb differences were no longer significant at that stage, and return to sport typically occurs later [36].

Considering the high rate of contralateral injuries in pediatric patients, the significantly better postural stability of the healthy limb in the LET group beyond 6 months may have clinical relevance. Better postural stability has been associated with a reduced risk of knee injury [21]. As reinjury remains a major concern in this population, this finding—if confirmed in studies with greater statistical power—could have important implications for rehabilitation practices, particularly regarding protection of the contralateral limb.

Although the BSS has been used in various medical fields, no validated GSI thresholds from BSS testing currently exist in the literature to guide return-to-sport decisions for pediatric or adult patients who underwent ACLR [23,24,25,26,27,28]. Therefore, GSI values obtained in this study cannot be compared with those of other studies. Current return-to-sport criteria in the pediatric ACLR populations primarily rely on muscular isokinetic strength symmetry, hop test performance, and validated clinical scores [37,38]. Furthermore, recent evidence highlighted the growing importance of objective functional assessment tools, such as force plate-based testing, in evaluating recovery after ACLR [39]. The BSS has not yet been included in routine testing. However, postural stability objectively measured using the BSS can be useful in the global neuromuscular function assessment of athletes post-ACLR due to its ability to predict the risk of second ACL injury [21]. In this context, the GSI should be interpreted as a complementary neuromuscular control parameter rather than a standalone clinical decision-making tool. Postural stability may capture aspects of dynamic control that are not fully assessed by strength or functional hop tests.

This study paves the way for the increased use of the BSS, allowing clinicians to access more information in order to make better-informed decisions regarding the return to sports.

This study does have its limitations. First, the retrospective design limits control over potential confounding factors. No a priori sample size calculation was performed due to the retrospective design of the study, which may limit statistical power and should be considered when interpreting the results. The study only included 100 patients because more than half of the patients screened did not have BSS assessments available in their electronic medical records. The BSS is unfortunately not routinely used in ACLR follow-up. Subgroup analyses reduced the number of patients and therefore statistical power, increasing the risk of type II error. The single-center design contributed to the limited sample size, while also limiting the generalizability of the results. Consequently, results should be interpreted with caution, and larger prospective cohorts are required to confirm these findings. Nevertheless, all eligible patients were included. The study is subject to selection bias. Comparison of the excluded and included study groups showed statistically significant differences for age and BMI. However, even if statistically significant, these differences are extremely small and are therefore not clinically significant. Median age in years with interquartile range is 16 (15–17) for included patients compared to 15 (10–18) for excluded ones. The difference is even smaller for the median BMI of 22.7 (21.2–26.2) for included patients compared to 22.2 (19.8–24.4). The other metrics did not differ significantly between the groups. Therefore, it is possible to confirm that the included and excluded groups are clinically comparable, which means the selection bias risk is minimal. Another limitation would be that no multivariable adjustment for potential confounders (age, sex, pivot shift grade, meniscal injury, and graft diameter) was performed. Additionally, this study did not make correlations between GSI and traditionally used ACLR functional scores. Furthermore, BSS assessments were conducted by different physiotherapists, which may have introduced inter-rater variability in test administration, potentially affecting the consistency of the GSI measurements obtained. Because LET performance became increasingly used after 2024, a temporal confounding factor may have been introduced. The use of LET was not randomly distributed over time, as it was implemented more recently. This means changes in surgical techniques, surgeon experience and rehabilitation protocols may have influenced outcomes. However, regression analyses demonstrated that there was no significant relationship between BSS performance and surgery date. Other limitations include that there was no measure of graft rupture rate, no clinical outcome scores, no return-to-sport data, and no assessment of rotational stability. Only level four of the platform was used for BSS assessments, meaning other levels might show differences. The method for handling patients with multiple BSS assessments for the same time period introduced potential bias toward better performers. The absence of exclusion of patients with prior contralateral knee surgery may represent another source of bias. Finally, whether patients touched the railing with their hands or the ground with their non-weight-bearing limb was not analyzable.

## 5. Conclusions

In this pediatric cohort undergoing ACLR with hamstring autograft, no statistically significant differences in postural stability were observed between patients with and without LET before and after 6 months postoperatively. Greater attention to the healthy limb during early rehabilitation may be warranted. Further studies with larger sample sizes are needed to confirm these findings.

## Figures and Tables

**Table 1:** Overall patient demographics and surgical information.

*n* = 100 *	
Age (years), Median [IQR] (min;max)	16 [15;17] (13;20)
Female, *n* (%)	54 (54%)
Right, *n* (%)	54 (54%)
BMI, Median [IQR] (min;max)	22.7 [21.2;26.2] (17.2;44.0)
Preoperative pivot shift Grade 1, *n* (%)	11 (11%)
Preoperative pivot shift Grade 2, *n* (%)	41 (41%)
Preoperative pivot shift Grade 3, *n* (%)	47 (47%)
LET, *n* (%)	35 (35%)
Medial meniscal lesion, *n* (%)	13 (13%)
Lateral meniscal lesion, *n* (%)	40 (40%)
Bilateral meniscal lesion, *n* (%)	11 (11%)
Femoral tunnel diameter (mm), Median [IQR] (min;max)	8.5 [8.0;9.0] (7.0;11.0)
Tibial tunnel diameter (mm), Median [IQR] (min;max)	8.5 [8.0;9.0] (7.0;11.0)

* One pivot shift value missing.

**Table 2:** Group comparison for patients with and without lateral extra-articular tenodesis (LET).

	Without LET (*n* = 65)	With LET (*n* = 35) *	*p*-Value **
**Age (years),** Median [IQR] (min;max)	16 [15;17] (13;20)	15 [15;16] (13;17)	0.062
**Female sex**, *n* (%)	32 (49.2%)	22 (62.9%)	0.192
**Right side**, *n* (%)	37 (56.9%)	17 (48.6%)	0.424
**BMI**, Median [IQR] (min;max)	22.9 [21.1;27.0] (17.2;44.0)	22.5 [21.3;24.1] (17.3;39.8)	0.389
**Preoperative pivot shift**, *n* (%)			0.068
Grade 1 «Glide»	11 (16.9%)	0 (0.0%)	
Grade 2 «Clunk»	26(40.0%)	15 (44.1%)	
Grade 3 «Gross/Locking»	28 (43.1%)	19 (55.9%)	
**Femoral tunnel diameter (mm)**, Median [IQR] (min;max)	8.5 [8.0;9.0] (7.0;10.5)	8.5 [8.0;9.0] (7.0;11.0)	0.643
**Tibial tunnel diameter (mm)**, Median [IQR] (min;max)	8.5 [8.0;9.0] (7.0;11.0)	8.5 [8.0;9.0] (7.0;11.0)	0.192
**Meniscal lesion**, *n* (%)			0.269
Medial	11 (16.9%)	2 (5.7%)	
Lateral	23 (35.4%)	17 (48.6%)	
Both	6 (9.2%)	5 (14.3%)	
None	25 (38.5%)	11 (31.4%)	

* One pivot shift value missing. ** For scale and ordinal variables, the Mann–Whitney U test was used. For nominal variables, the Chi-square or Fisher’s exact test was used.

**Table 3:** GSI for ACLR and ACLR plus LET groups. GSI, Global Stability Index; ACLR, Anterior cruciate ligament reconstruction; LET, Lateral extra-articular Tenodesis *.

	Without LET	With LET	Effect Size (95% CI) **	Mean Difference 95% CI	*p*-Value ***
**Before 6 months**	*n* = 42	*n* = 23			
Healthy leg	3.96 ± 1.86	3.07 ± 1.43	0.52 (0.00;1.03)	0.00;1.78	0.051
Operated leg	3.10 [2.18;4.38]	2.50 [2.00;3.20]	0.11 (−0.38;0.18)		0.372
**After 6 months**	*n* = 33	*n* = 16			
Healthy leg	3.86 ± 1.76	2.71 ± 0.88	0.75 (0.13;1.36)	0.39;1.91	0.004
Operated leg	3.10 [2.35;4.40]	2.90 [2.15;3.93]	0.11 (−0.18;0.13)		0.424

* Results are presented as Mean ± SD for the healthy leg, and median [IQR] for the operated leg. ** Cohen’s D for healthy leg and r for operated leg. *** *p*-value for the independent *t*-test (healthy leg) and Mann–Whitney U test (operated leg).

**Table 4:** Comparison of Global Stability Index between healthy and operated legs across postoperative time periods *.

	Healthy Leg	Operated Leg	*p*-Value
**Before 6 months (*****n*** **= 65)**	3.50 [2.30;4.60]	2.60 [2.10;3.85]	0.003
**After 6 months (*****n*** **= 49)**	3.20 [2.25;4.40]	3.00 [2.30;4.30]	0.945
**All together (*****n*** **= 100)**	3.10 [2.30;4.20]	2.65 [2.20;3.98]	0.059

* Results presented as Median [IQR].

**Table 5:** Group comparison for patients included and excluded from final analysis.

	Patients Included (*n* = 100)	Patients Excluded (*n* = 129)	*p*-Value *
**Age (years),** Median [IQR] (min;max)	16 [15;17] (13;20)	15 [10;18] (14;16)	0.026
**Female sex**, *n* (%)	54 (54.0%)	62 (48,8%)	0.504
**Right side**, *n* (%)	54 (54.0%)	65 (51.2%)	0.69
**BMI**, Median [IQR] (min;max)	22.7 [21.2;26.2] (17.2;44.0)	22.2 [19.8;24.4] (13.4;34.5)	0.015
**Preoperative pivot shift**, *n* (%)			0.469
Grade 1 « Glide »	11 (11.1%)	12 (9.9%)	
Grade 2 « Clunk »	41 (41.4%)	56 (46.3%)	
Grade 3 « Gross/Locking»	47 (47.5%)	51 (42.1%)	
**Femoral tunnel diameter (mm)**, Median [IQR] (min;max)	8.5 [8.0;9.0] (7.0;11.0)	8.5 [8.0;9.0] (7.5;10.5)	0.921
**Tibial tunnel diameter (mm)**, Median [IQR] (min;max)	8.5 [8.0;9.0] (7.0;11.0)	8.5 [8.0;9.0] (7.5;10.5)	0.714
**Meniscal lesion**, *n* (%)			0.24
Medial	13 (13.0%)	24 (18.6%)	
Lateral	40 (40.0%)	41 (31.8%)	
Both	11 (11.0%)	23 (17.8%)	
None	36 (36.0%)	41 (31.8%)	

* For scale and ordinal variables, Mann–Whitney U test was used. For nominal variables, Chi-square or Fisher’s exact test was used.

## Data Availability

The raw data supporting the conclusions of this article will be made available by the authors on request.

## Abbreviations

The following abbreviations are used in this manuscript:

ACL	Anterior cruciate ligament
LET	lateral extra-articular tenodesis
ACLR	anterior cruciate ligament reconstruction
BSS	Biodex Stability System
GSI	global stability index
LEAP	lateral extra-articular procedures
ALL	anterolateral ligament
